# May the force be with you

**DOI:** 10.7554/eLife.36662

**Published:** 2018-04-24

**Authors:** Shinuo Weng, John B Wallingford

**Affiliations:** Department of Molecular BiosciencesUniversity of Texas at AustinAustinUnited States

**Keywords:** biomechanics, force, convergent extension, gastrulation, blastopore closure, convergent thickening, *Xenopus*

## Abstract

Understanding the coordination of the forces generated in embryos by two processes, convergent extension and convergent thickening, is key to understanding how a hollow sphere of cells develops into an elongated embryo.

**Related research article** Shook DR, Kasprowicz EM, Davidson LA, Keller R. 2018. Large, long range tensile forces drive convergence during Xenopus blastopore closure and body axis elongation. *eLife* **7**:e26944. doi: 10.7554/eLife.26944

Gastrulation is a key embryonic event during which the three-dimensional framework of an animal is established. Indeed, it has been said that ‘it is not birth, marriage or death, but gastrulation which is truly the most important time in your life’ (Slack, 1991). Generally speaking, the process of gastrulation accomplishes two goals. First, it positions the precursors of the internal organs inside the embryo and surrounds them with cells that will go on to form skin. Second, it converts an essentially spherical ball of cells into the elongated form with the defined head-to-tail axis that is found in animals as diverse as insects, worms and vertebrates. Not surprisingly, achieving these two goals involves the robust movement of cells and tissue.

After more than a century of study, well-defined maps of these tissue movements have been established for many animals (Stern, 2004), and more recent work has focused on understanding how the forces responsible for tissue movement are generated and transmitted in a coordinated manner between individual cells.

A popular model for the study of gastrulation and tissue mechanics is the frog *Xenopus laevis* (Figure 1A; Keller et al., 2003). The large size of *Xenopus* cells makes them amenable to live imaging, and the even distribution of yolk throughout all the cells allows *Xenopus* tissue to survive and develop well in explant cultures (Davidson and Keller, 2007). However, the mechanisms by which the forces that move tissues are generated and directed have remained unclear. Now, in eLife, David Shook of the University of Virginia and co-workers – Eric Kasprowicz (Thomas Jefferson University Hospital), Lance Davidson (University of Pittsburgh) and Raymond Keller (Virginia) – report new insights into the generation of long-range forces during gastrulation in *Xenopus* (Shook et al., 2018).

**Figure 1.**
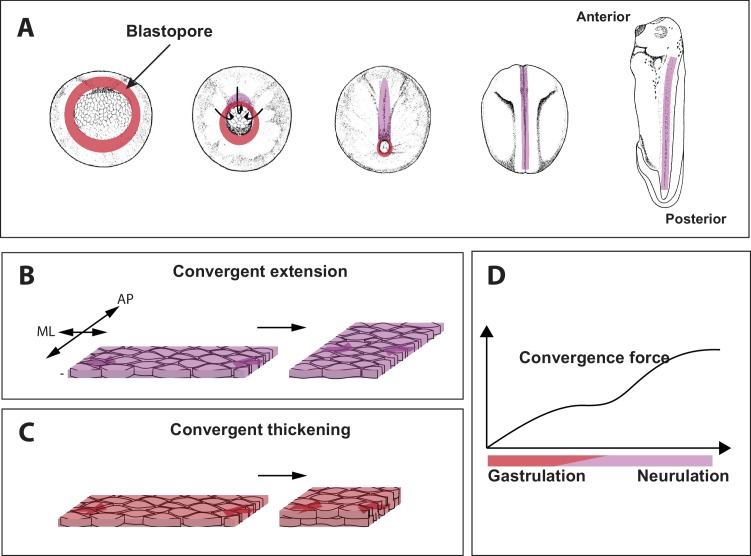
Force generating 'engines' during gastrulation in *Xenopus*. (**A**) During gastrulation a hollow sphere of cells (left) is transformed into an elongated embryo (right). This starts with cells on the dorsal (upper) side of the embryo rolling over a structure called the blastopore (red circle) and moving to the inside of the sphere. This reduces the size of the blastopore. Cell-autonomous convergence forces (purple) then cause the sphere to elongate, leading to the formation of the anterior–posterior axis. (**B**) The process by which tissue elongates along the anterior-posterior (AP) axis, and becomes narrower along the medio-lateral (ML) axis, is called convergent extension. (**C**) Shook et al. discovered that a process called convergent thickening – which involves the tissue becoming thicker in the direction at right angles to the convergent extension – is also important during gastrulation. (**D**). Sketch showing how the convergence force (y-axis) increases through gastrulation, and then plateaus (during early neurulation) before increasing again (during late neurulation).

Very early embryos are made up of three germ layers: the outer layer or ectoderm, the middle layer or mesoderm, and the inside layer or endoderm. During gastrulation in *Xenopus*, a ring of cells that later forms the mesoderm (called the presumptive mesoderm) rolls inwards over a structure called the blastopore lip (Figure 1A). This process is called involution. The presumptive mesoderm cells then rearrange to form a narrower and longer embryo in a process called axis elongation (Figure 1B; Shindo, 2018). Convergent extension has long been considered to be the sole source of convergence force in embryos and has been well characterized in animals ranging from insects to amphibians, fish and mammals.

Shook et al. identified a central role for another process, called convergent thickening, in which the cells rearrange themselves in order to increase the thickness of the tissue (Figure 1C). Curiously, convergent thickening had been identified decades ago, but was previously thought to play only a relatively minor role in gastrulation (Keller and Danilchik, 1988). Shook et al. found that the entire ring of presumptive mesoderm undergoes convergent thickening before involution, thus adding new layer of mechanical complexity to this long-studied process.

So how do embryos co-ordinate the processes of convergent extension and convergent thickening? The transition from convergent thickening before involution to convergent extension after involution suggests that there is a mechanical connection between these two processes that generates a driving force across the whole embryo throughout gastrulation. To explore this question Shook et al. used an experimental set-up called the 'tractor pull' to study samples of tissue taken from the presumptive mesoderm. This approach involves attaching two plastic strips to the ends of the tissue to control its position, and an optical fiber to pull the tissue; forces generated in the tissue cause the optical fiber to bend, and its deflection can be used to calculate the force.

Shook et al. found that the convergence force increased throughout gastrulation, then plateaued during a stage known as early neurulation (during which the central nervous system is formed), and then increased again during late neurulation (Figure 1D). Since convergent extension occurs dominantly in the dorsal tissue (that is, the upper side or back of the animal), and convergent thickening lasts longer on the ventral side (the lower side or front), Shook et al. tried to unravel the origin of the behavior shown in Figure 1D by changing the proportion of ventral and dorsal tissue. When just ventral tissue was studied, the second increase during late neurulation did not happen; and when just dorsal tissue was studied, there was no plateau phase. This suggests that the convergence force before involution is mostly driven by convergent thickening across the whole tissue, and that after involution convergence force results solely from convergent extension in the dorsal tissue.

Shook et al. also performed experiments in which the tractor pull set-up was used to apply forces to tissue. They found that dorsal tissues under convergent extension are capable of generating and transmitting higher forces than tissues under convergent thickening. Therefore, when the transition from thickening to extension occurs in the dorsal tissue and spreads to the ventral tissue, regions of ventral tissue that have not yet undergone thickening-to-extension transition will limit the generation and transmission of force in the medio-lateral direction.

The work of Shook et al. provides new insights into the mechanics of gastrulation, while raising new questions and invigorating old ones. For example, while the mechanisms of convergent extension are well defined (Shindo, 2018), the mechanisms that drive convergent thickening remain essentially unknown. Since convergent thickening relies on radially-directed cell movement, it is tempting to speculate that it uses similar mechanisms to other recently defined radial migration events (Sedzinski et al., 2016; Szabó et al., 2016). Moreover, while planar cell polarity signaling, a conserved genetic network that controls the position of cells within a population, is known to direct convergent extension (Butler and Wallingford, 2017), its role in convergent thickening is unknown; this issue warrants further examination because the disruption of planar cell polarity in ventral tissues where convergent thickening predominates substantially disrupts gastrulation (Ewald et al., 2004).

Ultimately, this work is significant because gastrulation in most animals relies on the complex interplay of multiple force-generating 'engines' (such as convergent extension and convergent thickening) yet how such engines work in concert remains largely unexplored in other animals, including mammals (Nowotschin and Hadjantonakis, 2010; Solnica-Krezel, 2005). The results reported by Shook et al. should motivate others to attack similarly complex processes in other animals.
